# Improving communication and practical skills in working with inpatients who self-harm: a pre-test/post-test study of the effects of a training programme

**DOI:** 10.1186/1471-244X-14-64

**Published:** 2014-03-04

**Authors:** Nienke Kool, Berno van Meijel, Bauke Koekkoek, Jaap van der Bijl, Ad Kerkhof

**Affiliations:** 1 Intensive Treatment Centre, Palier, The Hague, The Netherlands; 2 Department of Health, Sports & Welfare/Cluster Nursing, Research Group Mental Health Nursing, Inholland University of Applied Sciences, Amsterdam, The Netherlands; 3 Parnassia Academy, Parnassia Psychiatric Institute, The Hague, The Netherlands; 4 Research group for Social Psychiatry & Mental Health Nursing, HAN University of Applied Sciences, Nijmegen, The Netherlands; 5 ProCES, Pro Persona GGZ, Wolfheze, The Netherlands; 6 Department of Clinical Psychology, and EMGO+, Institute for Health and Care Research, VU University, Amsterdam, The Netherlands

**Keywords:** Self-harm, Training programme, Lay experts, Art, Psychiatry, Effects

## Abstract

**Background:**

Differing perspectives of self-harm may result in a struggle between patients and treatment staff. As a consequence, both sides have difficulty communicating effectively about the underlying problems and feelings surrounding self-harm. Between 2009 and 2011, a programme was developed and implemented to train mental health care staff (nurses, social workers, psychologists, psychiatrists, and occupational therapists) in how to communicate effectively with and care for patients who self-harm. An art exhibition focusing on self-harm supported the programme. Lay experts in self-harm, i.e. people who currently harm themselves, or who have harmed themselves in the past and have the skills to disseminate their knowledge and experience, played an important role throughout the programme.

**Methods:**

Paired sample t-tests were conducted to measure the effects of the training programme using the Attitude Towards Deliberate Self-Harm Questionnaire, the Self-Perceived Efficacy in Dealing with Self-Harm Questionnaire, and the Patient Contact Questionnaire. Effect sizes were calculated using *r*. Participants evaluated the training programme with the help of a survey. The questionnaires used in the survey were analysed descriptively.

**Results:**

Of the 281 persons who followed the training programme, 178 completed the questionnaires. The results show a significant increase in the total scores of the three questionnaires, with large to moderate effect sizes. Respondents were positive about the training, especially about the role of the lay expert.

**Conclusion:**

A specialised training programme in how to care for patients who self-harm can result in a more positive attitude towards self-harm patients, an improved self-efficacy in caring for patients who self-harm, and a greater closeness with the patients. The deployment of lay experts is essential here.

## Background

Self-harm can be defined as any intentional, direct or indirect harm of body tissue with a non-fatal outcome [1]. According to the literature, 4% of the general population [2] and 20% to 30% of the psychiatric population harm themselves in their lifetime [3]. The actual percentage is probably higher: people who harm themselves do not always seek help and not all self-harming behaviour is recorded [3-6]. There is increasing awareness that self-harm serves a purpose for patients. It supports them to regulate intense and overwhelming emotions and thoughts. For many self-harm patients, who often have a negative self-image and body experience, possibly caused by previous trauma and abuse, self-harm operates as a survival mechanism [7,8].

Mental health care staff are confronted with self-harm patients on a regular basis and react in a variety of ways, ranging from understanding and empathy to frustration, anger and powerlessness [9,10]. Most of their interventions focus on stopping the self-harming behaviour.

Generally, treatment staff find it difficult to build a professional relationship with patients who self-harm [11,12]. Many of them are insecure about their ability to deal effectively with self-harm, in addition to experiencing feelings of misunderstanding and frustration [7]. As a result, treatment staff can lose empathy towards patients and their reaction may be driven by countertransference. Several guidelines concerning the treatment of self-harming patients describe the necessity of connecting with the patients and understanding what self-harm means to them. In their contact with patients who self-harm, the treatment staff should focus on understanding and validating the patients’ feelings, and on meeting their specific needs [13-15].

Many patients who harm themselves are dissatisfied with mental health care [16,17]. According to Taylor et al*.* (2009), patients perceive a lack of knowledge, as well as negative attitudes and inappropriate behaviour among staff, such as being made to wait for hours before receiving treatment. Furthermore, patients participate insufficiently in decisions about their own treatment [17]. As a consequence, patients feel misunderstood and frustrated. In some cases this results in increased self-harming behaviour [7].

The different perspectives of self-harm may result in a struggle between patients and treatment staff. In this situation, both sides are unable to communicate effectively about the problems and feelings underlying self-harm. In order to respond to this problem, a programme has been developed to train mental health care staff in how to communicate with and care effectively for patients who self-harm^a^. The programme was carried out between 2009 and 2011 in the Netherlands, and was supported by an art exhibition focusing on self-harm. The aim of this article is to describe the scientific evaluation of this training programme.

## Methods

### Design

A quasi-experimental pre-test/post-test design was used to investigate the effects of the training programme. To check the stability of scores over time without any intervention, we carried out two pre-intervention measurements among three participating groups of the total sample with an intermediate period of ten weeks. The analyses of these measurements revealed no significant differences in terms of attitude, self-efficacy and distance-closeness scores, with p-values indicating a high stability.

### Participants

The programme was executed in eight mental health centres and one forensic-psychiatric centre across the Netherlands. In each centre, training was provided to forty employees. By the end of the programme, a total of 360 healthcare providers had been trained. Prior to the first measurement, the participants received a letter containing detailed information about the study, information about the questionnaires, and instructions on how to complete them. The letter explained that anonymity was guaranteed. The act of returning the questionnaires served as consent for participation.

The training programme targeted treatment staff who were in close contact with self-harming patients in their daily work, and mental health nurses and social workers in particular. Entire teams were encouraged to participate in the programme as we assumed that the effects of the training programme would improve if professionals went through the learning process together, thereby developing a collaborative view of caring for self-harm patients. The programme management recruited the mental health centres by contacting key figures (such as psychiatrists, directors) at these centres. They, in turn, recruited the participating wards and individual participants for the training programme. Some centres selected special wards where self-harm was a recurring problem, while others made the training available for all employees. One centre assigned nurses who were already attending an internal course.

### The intervention

The training programme is the result of cooperation between the Dutch Self-Harm Foundation (an organisation of lay experts), Inholland University of Applied Sciences, and Het Dolhuys—the Dutch national museum of psychiatry. Lay experts, i.e. people who currently harm themselves, or who have harmed themselves in the past and have the skills to disseminate their knowledge and experience, played an important role throughout the training programme. They worked in close cooperation with professionals to develop and implement the programme. Its basic principle was straightforward: in order to communicate with and care for patients who self-harm, staff need to understand the patients’ emotions, the difficulties they have in dealing with their emotions, and the relationship between emotions and self-harming behaviour. Staff also need to understand their own feelings and thoughts about self-harm and how these influence their reactions. A non-judgmental response is necessary in order to connect with self-harm patients. Connection between staff and the patient forms the basis of a supportive therapeutic relationship marked by clear and constructive communication.

The training programme was supported by an art exhibition. The language of art was used to express the deeper meaning of self-harm, for which words sometimes seem inadequate. The art objects were made by artists with hands-on self-harm experience. Most objects expressed feelings of intense emotional pain and loneliness, which are the emotions that occur most frequently in patients who harm themselves. The idea was that healthcare staff who visited the exhibition would become more sensitive to what self-harm means to the patients. The exhibition ran for several weeks prior to the training programme in a central location at each participating centre. The art objects were also available in a PowerPoint presentation which was used during the training programme. This was particularly valuable for participants who had not visited the exhibition.

The training programme was provided by a lay expert and a trainer with a nursing background. It lasted one and a half days, with an interval of three weeks between the first and the second training day, allowing participants to practice what they had learned in the first day. The trainers discussed the meaning and motives of self-harm, using the art objects as visual aids. That was followed by a methodical approach to analysing self-harming behaviour and formulating possible interventions. An important tool was the intervention programme, ‘Caring for patients who self-harm: a nursing intervention programme’, which had been previously developed by Bosman and Van Meijel [7]. The programme was based on an extensive literature search [7,18], combined with programme reviews by professional and lay experts [7]. Participants were given tools to practise recognising the early signs of self-harm and developing and executing an early intervention plan as essential parts of the intervention programme. The lay expert brought her own, personal story into the training programme, thereby creating a lively illustration of the experiential world of people who harm themselves. For the content of the training, see Table 1: Content of the training.

**Table 1:** Content of the training

**Day 1 (full day)**	**Day 2 (half day)**		
- Introduction and acquaintance	- Looking back on day 1 and the period between day 1 and today		
○ Emotions and reactions, individually and within the team	○ Did anything change in the participant’s feelings about self-harm patients? How did this influence the participants’ attitudes and behaviour?		
○ Dilemmas	- Discussion of homework assignment:		
- Communication about self-harm	○ Early intervention plan: difficulties and dilemmas		
○ Discussion of the art objects	○ Team discussion: reactions of the team. Did it lead to a collaborative approach of self-harm?		
○ Lay expert’s experiences	- Discussion of practice situations		
- Theoretical knowledge about self-harm	- Role-playing to practise communication skills		
- Communication and building a relationship	- Evaluation and closure		
○ Cooperation			
○ Exploring needs and alternatives for self-harm			
- Communication and recognition of early signs			
○ Triggers of self-harm			
○ Early signs (thoughts, feelings, behaviours)			
- Communication and cooperation			
○ Early intervention plan			
- Evaluation and homework assignment			
○ Drawing up an early intervention plan with a patient			
○ Discussion of self-harm within the treatment team			

Additionally, ‘train-the-trainer’ meetings were organised to disseminate knowledge and implement the intervention strategy in the participating mental health centres. A maximum of four employees per centre received a complementary one-day training in the intervention programme and in didactic skills to share knowledge and practical skills effectively to trainees.

### Data collection

#### Measurements

The outcomes of interest in the current study were: (a) the attitude of treatment staff towards self-harm patients; (b) the self-efficacy of treatment staff in dealing with self-harm patients; and (c) the distance between self-harm patients and treatment staff resulting from the avoidance of contact and/or inappropriate professional behaviour.

The Dutch version of the Attitudes Towards Deliberate Self-Harm Questionnaire (ADSHQ) [11] was used to measure the attitude of staff. The ADSHQ has a total of thirty-three items, including items such as:

– I often feel helpless when dealing with the problems that deliberate self-harm patients have.

– Self-harm patients just clog up the system.

– Self-harm patients are victims of some other social problems.

The ADSHQ has four dimensions:

(1) Perceived confidence in assessing and referring self-harm patients (eight items)

(2) Dealing effectively with self-harm patients (six items)

(3) Empathic approach (five items)

(4) Ability to cope effectively with legal and hospital regulations that guide practice (six items).

The remaining eight items concern other aspects of attitudes towards self-harm, such as ‘Sometimes people self-harm because their cultural beliefs condone this practice when they are dealing with traumatic issues’.

Respondents were asked to score the statements on a four-point Likert scale ranging from ‘strongly disagree’ to ‘strongly agree’. The total scores of the ADSHQ range from 33 to 132, with a higher score referring to a more positive attitude towards self-harm. The original questionnaire was translated into Dutch and back-translated into English. Content validity was tested with researchers (N = 3). They discussed the original and translated items until a consensus was reached about the correct translation. Feasibility was tested with thirteen mental health nurses. Each nurse filled in the questionnaire individually, following which the questions and answers were discussed together. Based on this feasibility test, the final Dutch version of the ADSHQ was established. The original ADSHQ had acceptable psychometric properties [11] but a relatively low internal consistency (Cronbach’s alpha) of .42 for the total score. The separate dimensions, however, had acceptable scores: dimension 1: α = .71, dimension 2: α = .73, dimension 3: α = .67, dimension 4: α = .57. In our study, Cronbach’s alpha for the total score was .62; for dimension 1: α = .40, dimension 2: α = .81, dimension 3: α = .49, dimension 4: α = .62.

Based on the literature about self-efficacy [19,20] and self-harm [7], we developed a new questionnaire to measure self-efficacy in caring for self-harm patients: the Self-Efficacy in Dealing with Self-Harm Questionnaire (SEDSHQ). The SEDSHQ consists of thirty-four items and measures whether staff perceive themselves capable of caring for patients who self-harm.

The SEDSHQ contains items such as:

– I think I am capable of seeing the purpose of the patient’s self-harming behaviour.

– I think I can recognise signals of imminent self-harm.

– I think I am able to talk about my own feelings about self-harm with colleagues.

This questionnaire also uses a four-point Likert scale ranging from ‘probably not’ to ‘definitely yes’. The total score ranges from 34 to 136, with a higher score indicating a higher level of perceived self-efficacy. Content validity was tested with experts (N = 8): seven mental health care experts in self-harm and one lay expert. They were asked to indicate for each item if it was ‘1 = irrelevant and should be deleted; 2 = unclear in its relevance because the meaning is unclear; 3 = relevant but in need of a minor adjustment; 4 = relevant and clearly formulated’. The instrument was revised accordingly. Feasibility was tested with nurses (N = 7), who did not express any problems understanding the questionnaire. In our sample, the internal consistency of the SEDSHQ was high: α = .95.

To measure the position of staff on the distance/closeness continuum in their relationship with patients, we used the validated Patient Contact Questionnaire (PCQ) [21]. Respondents could agree or disagree with statements on a nine-point scale ranging from ‘strongly disagree’ to ‘strongly agree’. The PCQ contains twenty statements about staff behaviour towards patients.

Examples of statements are:

– No matter how difficult the patients with whom I am in contact are, I do not withdraw from them.

– I can listen easily to the stories of depressed patients.

– With some patients I also discuss my own problems.

The total PCQ scores range from 20 to 180, where 180 indicates extreme proximity to the patient and 20 refers to great distance from the patient. According to Betgem (2000), the middle range of this continuum represents ‘detached concern’—the ability to create a balance between objectivity and showing emotional involvement in the patient [21]. The original PCQ had acceptable psychometric properties [21]. Internal consistency (Cronbach’s alpha) was .70. In our study the α was .80.

In addition to these instruments, data were collected on several background variables concerning the participants: gender, age, years of employment in psychiatry, education, experience with self-harm (professional and private) and whether the participant had had previous education in self-harm.

Finally, the participants’ training and learning experiences were evaluated. To this end, we used an evaluation form consisting of fifteen statements about learning effects, the organisation of the training, and the quality of the trainers. To respond to these statements, a five-point Likert scale was used ranging from ‘strongly disagree’ to ‘strongly agree’. We also asked two open-ended questions: (1) what training elements the participants considered essential, and (2) what the training had given them. Finally, participants could mention if there was anything they had missed during the training programme and if they had any additional comments.

#### Procedure of data collection

The study was carried out between 2009 and 2011. All participants in the training programme were requested to complete measurements at two points in time: a pre-test measurement two weeks before the start of the training programme, and a post-test measurement four weeks after the last training session. We sent the ADSHQ, SEDSHQ and PCQ to the participants by email or post. They completed the evaluation form, including background variables, at the end of the training during the last meeting.

### Ethical considerations

Since there were no patients involved in the research, review and approval by the Ethics Committee was not necessary according to Dutch legislation.

### Data analysis

A paired sample t-test was performed to test the differences between the pre- and post-test. Bonferroni corrections for multiple testing were applied by dividing the α value of 5% by the number of tests (n = 3) yielding a significance level of .017. Effect sizes (*r*) were calculated, where *r* = .10 refers to a small effect, *r* = .30 to a medium effect and *r* = .50 to a large effect [22]. Standard multiple regression was used to explore the role of the background variables on the outcomes.

Before analysing the data, we performed a missing values analysis. Questionnaires containing more than 20% missing values were deleted. In questionnaires with less than 20% missing values, the method of ‘case mean substitution’ was used to replace the missing value. According to Shrive et al. [23] this method can be used up to 30% missing items.

The mean was calculated for the statements of the evaluation form. The open questions were subjected to open coding leading to categories for the central themes that emerged from the qualitative data.

SPSS, version 20 for Windows (SPSS, Chicago, IL, USA) was used to perform the statistical analysis.

## Results

### Background variables

Three hundred and sixty persons started the training and a total of 281 persons completed it (78%). Reasons for dropping out included obligations in patient care, sickness, and resignation. One hundred and seventy-eight participants completed one or more questionnaires (49%). The professional backgrounds of the participants are shown in Table 2. The average age of the respondents was 38 years. They had worked an average of twelve years in general or forensic psychiatry. Most of the respondents (82%) had professional experience with self-harm, 19% of the respondents also had experience with self-harm in their personal lives (either themselves, family or friends). Only 4% of the respondents had had previous specialised training in the field of self-harm.

**Table 2:** Professionals backgrounds of the respondents

**Education**	**Number (%)**
Certified nurse assistant	4 (2.3)
Registered nurse	86 (48.4)
Social worker	31 (17.4)
Master of science in nursing/healthcare	4 (2.2)
(Clinical) psychologist/psychotherapist	12 (6.7)
Psychiatrist	1 (0.6)
Occupational therapist	13 (7.3)
Others	5 (2.8)
Unknown	22 (12.3)
**Total**	**178 (100)**

### Primary outcomes

The total score of the ADSHQ (attitude) increased significantly towards a more positive attitude to self-harm (t = -7.84, df = 170, p < .000, *r* = .52). Looking specifically at the four dimensions of the ADSHQ, we found a significant increase between pre- and post-test scores in three of the four dimensions (see Table 3). This means that, after the training programme, the respondents considered their approach to be more empathic and they perceived themselves better able to assess, refer, and take care of self-harm patients. Dimension four (effectively coping with legal and hospital regulations) showed no significant difference between the two measurements. The effect size of the total ADSHQ score indicated a large effect, as was the effect on dimension two of this scale (effectively dealing with self-harming patients). Dimensions one (assessment and referral) and three (empathic approach) showed a moderate to small effect size. There was no effect visible on dimension four.

**Table 3:** Pre-test/post-test results on outcome measurements (paired t-tests and effect sizes)

	**Pre-test**		**Post-test**					
	**N**	**M**	**SD**	**M**	**SD**	***t*-statistic**	**p (2-tailed)**	***r***
**ADSHQ total** (attitude)	171	95.56	5.14	98.67	5.89	-7.84	.000	.52
**ADSHQ 1** (assessment and referral)	171	22.79	2.00	23.29	2.02	-3.09	.002	.23
**ADSHQ 2 **(dealing effectively)	171	16.60	2.30	18.11	1.90	-9.51	.000	.59
**ADSHQ 3 **(empathic approach)	171	16.33	1.52	16.80	1.62	-3.81	.000	.28
**ADSHQ 4 **(legal and hospital regulations)	171	17.85	2.11	17.54	2.60	1.85	.066	.14
**SEDSHQ** (self-efficacy)	174	92.59	16.61	101.77	15.73	-8.55	.000	.55
**PCQ** (patient-contact)	175	120.27	13.41	124.29	13.82	-5.45	.000	.38

ADSHQ: Attitudes towards Deliberate Self-Harm Questionnaire.

SEDSHQ: Self-Efficay in Dealing with Self-Harm Questionnaire.

PCQ: Patient Contact Questionnaire.

The scores on the SEDSHQ (self-efficacy) also showed a significant increase (t = -8.55, df = 173, p < .000, *r* = .55). This means that the respondents perceived their competence in dealing effectively with patients who self-harm to have increased after participating in the training programme. With an effect size of .55, the effect must be considered large.

We also found a significant change in the PCQ (detached concern) (t = -5.45, df = 174, p < .000), with a moderate effect size of .38. The figures indicate an attitudinal change of greater closeness to the patients who harm themselves. (see Table 3: Pre-test/post-test results on outcome measurements).

No significant associations were found between background variables (gender, age, years of employment in psychiatry, education, experience and previous education) and the training programme’s effects.

### Evaluation of the training

Generally speaking, trainees were satisfied about the course trainers, content and organisation. The combination of a lay expert trainer and professional trainer in particular was evaluated positively: this item has the highest score of all the statements. For detailed information about the trainees’ evaluations, see Table 4.

**Table 4:** Evaluation of the training

	**M**	**N**
**I found the combination of lay expert trainer and professional trainer instructive**	4.74	268
**I found the trainers qualified**	4.60	268
**The trainers complemented each other**	4.57	268
**The trainers answered the questions sufficiently**	4.54	268
**The attitude of the trainers stimulated me to reflect**	4.49	268
**Training was practical**	4.11	267
**I found the feedback day instructive**	4.10	267
**There was sufficient variety in the training methods**	3.99	267
**The training lived up to my expectations**	3.97	267
**The intervention programme is practical and useful**	3.94	264
**I behave differently with self-harm patients as a result of this course**	3.81	264
**I learned a lot by practicing communication skills**	3.67	206
**The length of the training was good**	3.62	268
**I found the homework useful**	3.46	235
**I need a follow-up training**	3.36	262

Note: meaning of the scores:

5 = totally agree, 4 = agree, 3 = neutral, 2 = disagree, 1 = totally disagree.

Concerning the question about the essential elements of the training, the trainees mentioned communication skills, a better understanding of patients, and a focus on cooperation with the patient. Some of the trainees reported that they felt less powerless because of their increased understanding of both the patients’ and their own behaviour and that understanding self-harming behaviour and the underlying problems and communicating about it with the patients were crucial. Furthermore, they had learned that exerting unilateral pressure on the patient to stop this behaviour was counterproductive in many cases. One of the respondents said, *‘I learned that you do not have to control the (self-harm) behaviour. Obviously, a patient needs that behaviour at that moment. This gives me peace’*.

As to the question of what the training gave them, most trainees mentioned knowledge, understanding, and acceptance. Overall the respondents stated that their perspective of self-harm had changed as a result of the training. There was greater emphasis on the patients' powerlessness instead of the assumed attention-seeking and manipulative intentions. They generally felt inspired and motivated to evaluate and change their personal and team perspectives of self-harm. About half of the respondents mentioned the value of practical tools for dealing with self-harm, particularly the structured intervention programme and working collaboratively with an early recognition plan.

Of all respondents, 73% (N = 268) did not miss any specific aspects regarding the training. The remaining 27% mentioned the length of the training most often: they thought the training was too short. Other aspects included a desire for more training in practical skills, more theoretical knowledge, greater attention to specific aspects during the training (such as caring for self-harming outpatients, the participation of parents of self-harming youths in treatment, addiction and self-harm, and the influence of self-harm on other patients on the ward). A few participants criticised the specific focus on nursing care and missed a multidisciplinary perspective.

As to the ‘train-the-trainer’ meetings, one important conclusion can be drawn from the evaluation: following the ‘train-the-trainer’ sessions, trainees remained insecure about their ability to continue the training in their setting. Some of the participants were experienced nurses who missed the didactic skills to provide training; other participants were skilled in education but missed practical knowledge and skills concerning caring for patients with self-harm.

## Discussion

We implemented and evaluated a training programme aimed at improving communication and practical skills in staff working with self-harm patients in inpatient settings. During both the development and implementation of the programme, we worked closely together with lay experts and focused intensively on communicating effectively with the patients. The art objects made by lay experts were a useful tool in expressing the emotions and motives of patients concerning self-harm. The personal story of the lay expert illustrated the patients’ perspective and, specifically, the crucial role of effective communication in treatment. The trainers emphasised the importance of communicating with empathy and understanding the meaning of self-harm for the patients.

Based on the results of this study, we can conclude that the training programme influenced the attitude and self-efficacy concerning self-harm positively. A significantly higher total score on the ADSHQ indicated a more positive attitude towards self-harm patients [11]. However, there were differences between the separate dimensions of this scale. Dimension two—dealing effectively with self-harm patients—showed the highest increase with a large effect size. Dimension three—empathic approach—also improved significantly, although with a moderate effect size. This means that participants were better able to understand and validate the feelings and thoughts of self-harm patients. Dimension one—confidence in assessment and referral—showed a small but significant change, and dimension four—coping effectively with legal and hospital regulations—showed no change. Actually, these last two dimensions were not the primary scope of the training. According to McAllister et al. staff are less likely to have a negative attitude if they perceive themselves as skilled to address the needs of patients who self-harm and, as a consequence, feel better equipped to work with such patients [11]. Attitude is a multidimensional construct with cognitive, affective, and behavioral components [24]. In this study, we followed the dimensions constructed by McAllister et al. [11], but there are without doubt other conceptual definitions of dimensions of attitude towards self-harm.

The significant increase in the scores on the SEDSHQ indicates that programme participants perceived an improved self-efficacy in caring for self-harm patients. This is consistent with the positive outcome in dimension two of the ADSHQ, which refers to the capabilities of professionals to deal effectively with patients who harm themselves. The results of our study are in line with other studies aiming at a more positive attitude on the part of staff towards self-harm patients. Several studies revealed that training professionals leads to a better understanding of self-harming behaviour and a more positive attitude towards these patients [25-27].

Concerning the position of the participants on the distance/closeness continuum (PCQ), we observed a shift towards closer contact with the patient. This is in line with the purpose of our project: we pursued a more empathic attitude on the part of healthcare staff towards the patient to improve their ability to understand the patients’ experiences, motives, emotions, and behaviour. However, it should be noted that this finding is open to interpretation. Too much closeness with the patient can lead to a situation in which the professional becomes overprotective and develops strong feelings of responsibility for all the patient’s ups and downs. As a consequence, the care can become suffocating for the patient and the great sense of responsibility increases the chance of a burn-out for the professional [28], especially when a patient does not recover soon enough. A balance between closeness and distance is necessary and must be found in every situation with every patient. The carer must be close enough to develop a good and empathic connection with the patient, and distant enough to prevent over-identification with the patient’s problems and the risk of burnout [21].

The evaluation of the training programme confirms the improved positive attitude of the trainees. Participants mentioned the focus on a better understanding of the patients’ emotions and behaviours combined with the focus on effective communication with the patients as crucial elements of the training programme. More than 70% stated that their interaction with patients had shifted as a result of the training, indicating that, not only did the training programme result in changed attitudes, but in changes in behaviour as well. According to Ajzen and Fishbein [24] people’s behaviour follows reasonably from their beliefs, attitudes and intentions. Our study does not confirm unambiguously whether the change in beliefs, attitudes and intentions actually led to behavioural changes among the participants and to a higher quality of care. Follow-up studies should focus on the actual behavioural changes and improvements in the quality of care for patients as a consequence of improved attitudes.

Of all participants in this study, only 4% had received previous training in the field of self-harm. Compared with other studies, where about 20% of the professionals had had specialised training in self-harm [11,29], this is a disturbing observation. Without focused training on self-harm, there is a risk of inadequate care for self-harm patients, which may lead to greater dissatisfaction and avoidance in care, resulting in an increase of morbidity and mortality [11]. As caring for patients with self-harm is mostly complex, professionals need to be sufficiently trained to ensure good quality of care.

### Limitations

There are several limitations in our study. One is the number of respondents who did not complete the questionnaires (51%). We do not know if these non-responders differ from the responders in terms of certain attitudinal aspects, thereby leading to response bias. The use of self-report questionnaires has to be mentioned as well, as this method increases the chance of socially desirable answers, especially concerning a sensitive theme like self-harm and the use of lay experts. Anonymity of the data may have reduced this effect. The absence of a control group is another limitation. However, the pre-intervention measurements prior to the training were stable over time in the absence of any intervention, which supports our assumption that the increased scores on our outcome measures can be attributed to the effects of the training. The internal consistency of the ADSHQ should be mentioned here as well. The low to moderate alphas for this instrument raises questions of its reliability. Although we used two other instruments with acceptable or good internal consistency, the results of our study must be interpreted with some caution. We advise further research into the psychometric aspects of the Dutch version of the ADSHQ. Further, we cannot be sure whether the effects of the training were achieved by the training's content or by other aspects, such as the art exhibition or the extra attention staff received when they followed the training. Results should therefore be interpreted with this limitation in mind.

A last but important limitation is the absence of any measured impact of the training on patient care. We recommend further research on the influence of training the treatment staff in communication and practical skills on the acceptance of care, treatment adherence, and level of self-harm of patients. We also advise a patient-centred study, where inquiries can be made into whether patients noticed an improved level of care after staff took a training course.

## Conclusion

In this study we presented the effects of a training programme for mental health care staff in how to communicate with and care for patients who self-harm. Participants in this study had a more positive attitude towards self-harm patients after taking the training programme. They perceived an improved ability to care for the patients and a better connection with the patients. Given the complexity of the care for self-harm patients, the low number of participants who had had prior training in self-harm was a worrisome finding. Self-harm patients need high-quality specialised care, and all healthcare staff need specialised training in the field of self-harm. Efforts should be made to integrate training in self-harm into basic medical, nursing, social work, and psychological education as well as in advanced professional courses.

## Endnote

^a^This study is based on the project “I’m doing just fine”. Saying “I’m doing just fine” is a common response among patients as a way of concealing their true state and avoiding further questioning by people in their social network.

## Competing interests

The authors declare that they have no competing interests.

## Authors’ contributions

NK conceived and developed the design of the study, collected the data, performed the statistic analysis and drafted the manuscript. BM, BK, JB, AK conceived and developed the design of the study, contributed to the interpretation of the data and in drafting the manuscript. All authors read and approved the final manuscript.

## Pre-publication history

The pre-publication history for this paper can be accessed here:

http://www.biomedcentral.com/1471-244X/14/64/prepub
